# General Strategy for Broadband Coherent Perfect Absorption and Multi-wavelength All-optical Switching Based on Epsilon-Near-Zero Multilayer Films

**DOI:** 10.1038/srep22941

**Published:** 2016-03-11

**Authors:** Tae Young Kim, Md. Alamgir Badsha, Junho Yoon, Seon Young Lee, Young Chul Jun, Chang Kwon Hwangbo

**Affiliations:** 1 Department of Physics, Inha University, Incheon 22212, Republic of Korea; 2 School of Materials Science and Engineering, Ulsan National Institute of Science and Technology (UNIST), Ulsan 44919, Republic of Korea

## Abstract

We propose a general, easy-to-implement scheme for broadband coherent perfect absorption (CPA) using epsilon-near-zero (ENZ) multilayer films. Specifically, we employ indium tin oxide (ITO) as a tunable ENZ material, and theoretically investigate CPA in the near-infrared region. We first derive general CPA conditions using the scattering matrix and the admittance matching methods. Then, by combining these two methods, we extract analytic expressions for all relevant parameters for CPA. Based on this theoretical framework, we proceed to study ENZ CPA in a single layer ITO film and apply it to all-optical switching. Finally, using an ITO multilayer of different ENZ wavelengths, we implement broadband ENZ CPA structures and investigate multi-wavelength all-optical switching in the technologically important telecommunication window. In our design, the admittance matching diagram was employed to graphically extract not only the structural parameters (the film thicknesses and incident angles), but also the input beam parameters (the irradiance ratio and phase difference between two input beams). We find that the multi-wavelength all-optical switching in our broadband ENZ CPA system can be fully controlled by the phase difference between two input beams. The simple but general design principles and analyses in this work can be widely used in various thin-film devices.

Coherent perfect absorption (CPA) occurs due to the interference of two counter-propagating beams, which leads to complete absorption of light in an absorbing medium^1^,^2^,^3^,^4^. It is also known as a time-reversed laser or an anti-laser. It was first demonstrated in a Fabry-Perot cavity with a wavelength-scale silicon slab. Incident light is trapped in a cavity, bouncing back and forth, until it is completely absorbed and converted to heat or other form of energy. Later, CPA was studied for various nanostructures and subwavelength thin films^5^,^6^,^7^,^8^,^9^,^10^,^11^, and was measured recently for graphene and conducting thin films^12^,^13^. However, the CPA structures so far are mostly limited to single-frequency operation, and are hard to be extended to a broadband structure - especially in the high, optical frequency region.

Here, we propose a new broadband CPA scheme based on epsilon-near-zero (ENZ) multilayer films. ENZ thin films can be a versatile platform for strong field confinement and for the enhancement of light-matter interactions^14^,^15^,^16^. Recently, it was found that unidirectional perfect absorption can occur in ultra-thin, low-loss ENZ layers^17^,^18^,^19^. Furthermore, at ENZ wavelengths (i.e. *Re*(*ε*) = 0), the normal component (E_⊥_) of the electric field can become very strong in a deeply subwavelength film (following the boundary condition ε_1_E_1⊥_ = ε_2_E_2⊥_). This strong field enhancement in ENZ films was also used to enhance nonlinear harmonic generation or control light absorption electrically in a cavity^20^,^21^. Interestingly, for very low-loss (Im(ε) ~ 0) dielectric films, it was reported that multilayers of high and low index films can be designed for unidirectional perfect absorption with field enhancement^22^.

In our previous theoretical and experimental works, we used ITO as a near-infrared (near-IR) ENZ material, and demonstrated perfect absorption in an ultra-thin ITO film. In these works, an ITO film was coated on a reflective substrate (or we worked in the attenuated total reflection condition) in order to suppress the transmission. In this case, by finding the destructive interference condition of reflected light, we could achieve *unidirectional* perfect absorption (i.e. having a single input beam)^23^. The ENZ wavelengths of ITO films depend on doping levels that can be controlled during the film growth, and thus tunable perfect absorption could be achieved without any structural patterning. Moreover, by using an ITO multilayer of different ENZ wavelengths, we experimentally demonstrated ultra-wideband perfect absorption in the near-IR^24^. Inspired by previous studies, here we propose a general strategy for broadband, *bi-directional* CPA based on planar, unpatterned films. Again, we use an ITO thin film as an ENZ layer, and find broadband CPA conditions in the near-IR region. Finally, we use this broadband ENZ CPA for multi-wavelength all-optical switching in the telecommunication window. In our analysis, we also fully account for the effects of the substrate. Therefore, we consider realistic configurations for our analysis. This method can be also readily extended to other frequency ranges using different ENZ materials (e.g. it can be extended to mid- and far-infrared using doped semiconductors). Our work shows that ENZ multilayer films provide a very flexible platform for high-frequency, broadband CPA.

In this paper, we first derive the CPA conditions using the scattering matrix and the admittance matching methods separately. Herein, we show that these two CPA analysis methods are equivalent. By combining these two methods, we extract not only the structural parameters of the CPA system, such as thin film thicknesses and incident angles, but also the input beam parameters, such as the amplitude ratio and phase difference between the two input beams. We also show that the broad ENZ wavelength band in the effective dielectric constant of the ITO multilayer film is important in achieving broadband CPA. Finally, we investigate multi-wavelength optical switching in our broadband CPA system consisting of an ITO multilayer sandwiched between two ZnSe prisms with 45° incident angles. It is also verified that our analytic calculations agree well with numerical simulations. The design principles and analyses used here can be widely applied to various nanophotonic devices and components, such as optical switches, modulators, filters, sensors, and thermal emitters.

## Theoretical Framework

### Scattering Matrix Method

**Figure 1:**
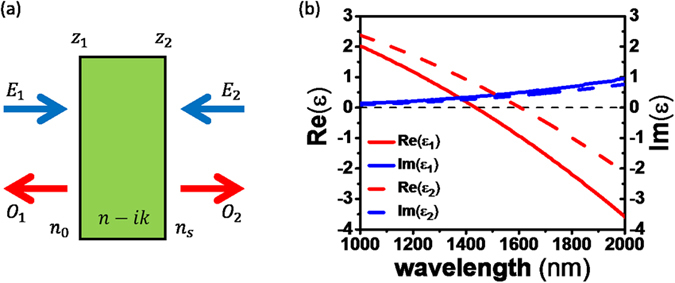
(**a**) General configuration of a two-port thin film system for CPA. *E*_1_, *E*_2_ and *O*_1_, *O*_2_ are input and output fields, respectively. An absorbing film with a complex refractive index (*N*_*f*_ = *n* − *ik*) with thickness *d* is located between two nonabsorbing media with refractive indices *n*_0_ and *n*_*s*_. (**b**) The dielectric constants of ITO-1 and ITO-2 films at near-IR wavelengths. The ENZ wavelengths (i.e. *Re*(*ε*) = 0) are 1435 nm for ITO-1 and 1605 nm for ITO-2.

We first obtain general conditions for CPA using the scattering matrix method. CPA occurs when the determinant of the scattering matrix of an absorbing medium becomes zero. This is a very general condition that can be applied to either a structured medium or an unpatterned film. The schematic diagram of CPA in an absorbing medium of complex refractive index *N*_*f*_ = *n* − *ik* (dielectric constant 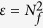) is shown in Fig. 1(a). The monochromatic time harmonic convention, *e*^*iωt*^, is used in this paper. The optical system consists of two ports; one on the left of the medium and the other on the right. Port 1 has the input and out fields *E*_1_ and *O*_1_ in the +*z* and −*z* directions, respectively, in the nonabsorbing incident medium of a refractive index *n*_0_. Similarly, Port 2 has the input and out fields *E*_2_ and *O*_2_ in the −*z* and +*z* directions, respectively, in the nonabsorbing substrate medium of a refractive index *n*_*s*_. The substrate (*n*_*s*_) and incident (*n*_0_) media are the output media for *E*_1_ and *E*_2_, respectively. Then the relationship between the output  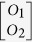  and input  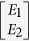  fields can be expressed using a scattering matrix as


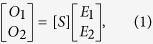


where the scattering matrix [*S*] is defined as


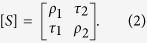


The elements of the scattering matrix are as follows: 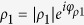 and 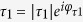 are the reflection and transmission coefficients, respectively, of the incident field *E*_1_, and 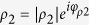 and 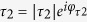 are the reflection and transmission coefficients, respectively, of the incident field *E*_2_^25^,^26^. The reflection phases are *φ*_*ρ*1_ and *φ*_*ρ*2_, and the transmission phases are *φ*_*τ*1_ and *φ*_*τ*2_.

Since CPA occurs when *O*_1_ = *O*_2_ = 0 (i.e. the determinant of scattering matrix [*S*] must be zero), the CPA condition is given by


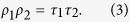


This can be, in turn, rewritten as


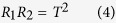


and


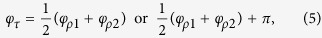


where 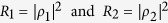. In deriving Eqs (4) and (5), the principle of the reversibility of 
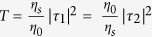
 and *φ*_*τ*1_ = *φ*_*τ*2_ were employed. The definitions of the admittances *η*_0_, *η*_*f*_, and *η*_*s*_ in each medium for the TE and TM waves are presented in Supplementary Information (Supplementary Note A)^26^,^27^.

By taking into account the amplitude ratio and phase difference between the two input beams, Eq. (1) can be expressed as


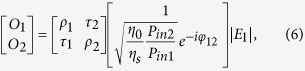


where *φ*_12_ = *φ*_2_ − *φ*_1_ is the phase difference between the two input beams, and the input irradiances at Port 1 and 2 are defined respectively as 
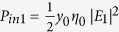
 and 
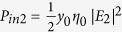
, where 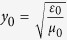 is the admittance of the vacuum. Then, the output irradiance *P*_*out*1_ in the incident medium of Port 1 can be written as


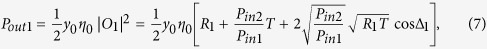


where Δ_1_ = *φ*_*ρ*1_ − *φ*_*τ*_ + *φ*_12_ is the interference phase, and we assumed |*E*_1_| = 1. Similarly, the output irradiance *P*_*out*2_ in the substrate becomes


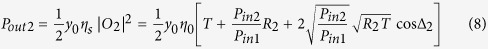


where 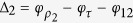.

Since *P*_*out*1_ = *P*_*out*2_ = 0 at CPA (i.e. complete destructive interference) occurs in Eqs (7) and (8), the input beam requirements, namely, the irradiance ratio and phase shift, can be respectively derived to be


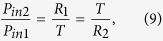


and


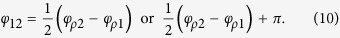


Equation (9), which is equivalent to Eq. (4), is related to balancing of the amplitudes between two output beams. Then, the output irradiances become


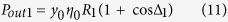


and


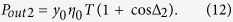


If *φ*_*ρ*2_ = *φ*_*ρ*1_ or *φ*_*ρ*1_ + π in Eq. (10) (i.e., if *φ*_12_ = 0 or π), then *P*_*out*1_ = *P*_*out*2_ = 0, which is “*two-port*” CPA. If *φ*_12_ = 0, the two input beams are called symmetric, whereas if *φ*_12_ = π, they are antisymmetric. Equations (11) and (12) indicate that CPA can be used as a sinusoidal optical switch or modulator if *φ*_12_ is varied. We may tune *φ*_12_ to make either *P*_*out*1_ = 0 or *P*_*out*2_ = 0, yielding “*one-port*” CPA. The output irradiances and absorptance (*A*) in the figures presented herein were normalized with respect to the total input irradiance (*P*_*in*1_ + *P*_*in*2_): 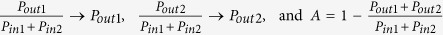.

### Admittance Matching Method

Now we consider CPA conditions using the admittance matching method that can be applied to unpatterned films. This will be eventually used in the next section when we design a near-IR CPA system with ITO multilayers.

When CPA occurs with *O*_1_ = *O*_2_ = 0 in Fig. 1, only one incident beam exists in each medium: *E*_1_ in the incident medium (*n*_0_) and *E*_2_ in the substrate (*n*_*s*_). Since the waves in the two media are counter-propagating, the magnetic field direction in the substrate should be reversed in the opposite direction to that in the incident medium (i.e., *H* →−*H*) if the electric field direction is the same. Then the admittance of the substrate can be defined as −*η*_*s*_ when CPA is present^28^. The negative admittance does not mean a negative index.

Therefore the forward admittance of the film starting from (−*η*_*s*_, 0) can be calculated using the transfer matrix method (TMM) as follows:


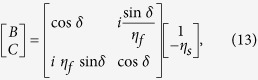


where *B* and *C* are the normalized electric and magnetic fields, respectively, in the incident medium, and the optical phase thickness is defined as 
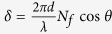
. And *N*_*f*_ = *n* − *ik*, *d*, and *η*_*f*_ are the complex refractive index of the film, physical thickness, and admittance of the thin film, respectively^26^. Snell’s law is valid here: 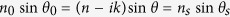, where *θ*_0_, *θ,* and *θ*_*s*_ are the incident angle, transmitted angle in the film, and transmitted angle in the substrate, respectively. The surface admittance of the film at *z*_1_ is given by


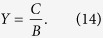


Then, CPA occurs when


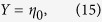


where *η*_0_ is the admittance of the incident medium. From Eqs (13) ~ (15), the CPA condition for an absorbing thin-film layer can be derived to be


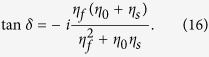


If the backward admittance matching is traced from *η*_0_ to −*η*_*s*_ using −*δ* in Eq. (13), the same CPA condition as Eq. (16) can be obtained. For a free-standing film in air at normal incidence, Eq. (16) is identical to Eqs (4, 7 and 14) in Refs 1, 7 and 8, respectively.

The structural parameters of thin films exhibiting CPA, such as their materials, thicknesses, and the incident light angles in the two media, can be determined from Eq. (16). They can be also obtained *graphically* using the admittance diagram^23^,^28^. In the next section, we will use the admittance diagram to determine the CPA parameters for ITO thin films. The elements of the scattering matrix in Eq. (2) are obtained in Supplementary Note A. It is also shown therein that Eq. (16), which was derived using admittance matching, is equivalent to Eq. (3), obtained from the scattering matrix. Thus, the relationship between the reflection and transmission coefficients can be expressed as


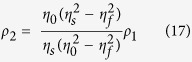


and


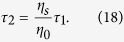


Then, the CPA condition of Eq. (3) for a single layer can be expressed as


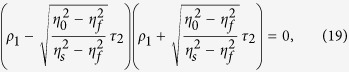


indicating that two input beams have even and odd phases. Also, the input beam parameters for single-layer CPA (more specifically, the irradiance ratio and phase shift given in Eqs (9) and (10)) can be respectively expressed by


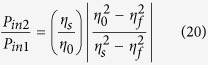


and


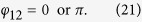


If a single absorbing layer cannot complete this admittance matching between the substrate and the incident medium, another phase-matching dielectric layer can be added to complete the trajectory of the admittance locus for Eq. (15) in the admittance matching diagram, in which case the phase shift of Eq. (21) is not necessarily 0 or π^28^.

In our previous work on unidirectional perfect absorption, we showed that the admittance matching condition (Eq. (15)) can be satisfied in an ultra-thin, low-loss ENZ film for p-polarized oblique incidence of light^23^,^24^. This can be directly extended to bi-directional CPA based on ENZ films, using the negative admittance for a substrate. Moreover, employing an ENZ multilayer film, we can achieve broadband CPA. This will be discussed more in the next section using ITO films.

ENZ CPA can be also understood as critical coupling, as explained in Supplementary Notes B and C. Since ENZ CPA is caused by resonant plasmon absorption, we can rewrite the admittance matching condition of Eq. (16) as the phase matching condition for transverse resonance in the film:


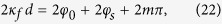


where *κ*_*f*_ is the transverse wavevector in the film, 2*φ*_0_ and 2*φ*_*s*_ are the reflection phases at the incident medium-film and film-substrate interfaces, respectively, and *m* is the order of a resonance mode. Eq. (22) indicates that once the critical coupling condition is satisfied for an ENZ film, two counter-propagating waves in the absorbing film interfere constructively, behave like an inhomogeneous guided mode in the film, and are totally absorbed as the mode propagates in the film.

## Results

The ENZ wavelengths of ITO films in the near-IR region can be controlled by doping levels. In our previous work, broadband perfect absorption with unidirectional illumination was experimentally realized using an ITO multilayer film^24^. In this work, we again use ITO as a tunable ENZ material. More specifically, we use two ITO films (ITO-1, ITO-2) as near-IR ENZ materials, whose ENZ wavelengths (i.e. *Re*(*ε*) = 0) are 1435 nm (ITO-1) and 1605 nm (ITO-2), as shown in Fig. 1(b) 29.

### ENZ CPA in a Single-layer ITO Thin Film and Its Application to All-optical Switching

**Figure 2:**
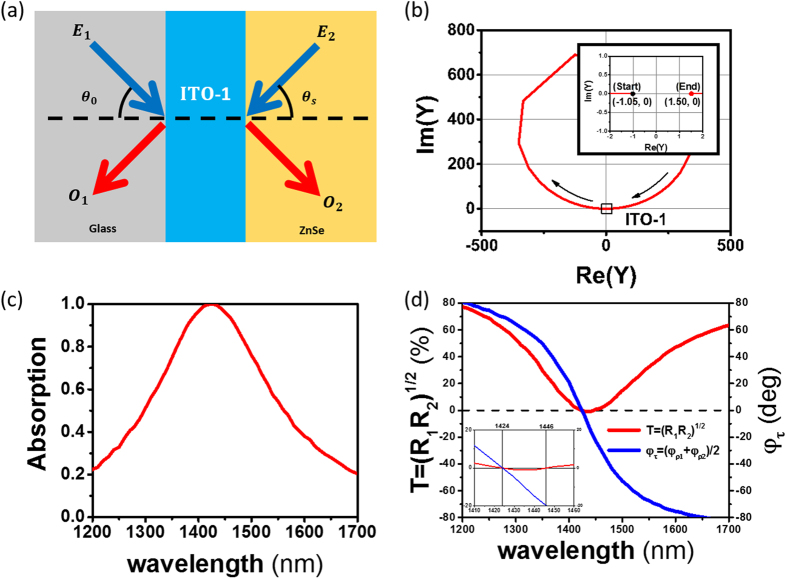
(**a**) Schematic diagram of ENZ ITO thin-film [Glass|ITO-1|ZnSe] CPA device; (**b**) the modified admittance diagram of [Glass|ITO-1|ZnSe] at 1424 nm wavelength, at oblique angles of 70° in glass (*n*_0_ = 1.50) and 35.2° in ZnSe (*n*_*s*_ = 2.45), and at 23.13 nm ITO-1 film thickness (*N*_*f*_ = 0.458 − *i*0.382) (inset shows start and end admittances); (**c**) calculated CPA spectrum of [Glass|ITO-1|ZnSe], *A* = 1 (CPA) is observed at 1424 nm; (**d**) calculated (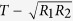) (red) and 
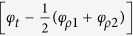
 (blue) of [Glass|ITO-1|ZnSe] as a function of wavelength (inset is expanded view near CPA wavelength regime); 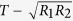 occurs at 1424 and 1446 nm whereas 
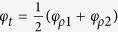
 occurs at 1424 nm.

We start with a CPA structure including a single ITO layer (ITO-1). We assume different materials for the incident and substrate media (i.e. an asymmetric structure), as shown in Fig. 2(a). We will first find CPA parameters graphically using the admittance matching diagram^26^,^28^,^30^, and then compare them with the general CPA conditions derived from the scattering matrix method.

The admittance diagram in Fig. 2(b) shows the admittance matching results for [Glass|ITO-1|ZnSe] at a wavelength of 1424 nm; the forward locus of the modified admittance of the ENZ ITO film starts at the negative modified admittance of (−1.05, 0) for the ZnSe substrate, makes a large circle in the clockwise direction corresponding to the film thickness of 23.13 nm, and arrives at a positive modified admittance of (1.50, 0) for the glass^28^. The incident angles were determined to be 70° in the glass and 35.2° in the ZnSe substrate.

The absorption spectrum of [Glassr|ITO-1|ZnSe] is calculated using the transfer matrix method (TMM), and is shown in Fig. 2(c), revealing CPA (*A* = 1) at 1424 nm, which is slightly shorter than the ENZ wavelength of 1435 nm. The same behavior was also observed in our previous study on unidirectional perfection absorption in ITO films^23^. It can be understood in this way: since CPA occurs due to critical coupling to a radiative mode in the ENZ thin film, CPA should follow the dispersion of this mode, which lies slightly above the ENZ frequency (*Re*(*ε*) = 0). Therefore, CPA occurs at a slightly shorter wavelength than the ENZ wavelength.

The elements of the scattering matrix in Eq. (2) were also calculated in Fig. 2(d). It can be seen that 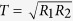 (red) occurs at 1424 nm and 1446 nm and that 
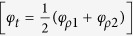
 (blue) occurs at 1424 nm. The CPA wavelength at 1424 nm, which satisfies both Eqs (4) and (5) and is shown in the inset of Fig. 2(d), was also used to obtain the admittance matching in Fig. 2(b), implying that Eq. (16) is equivalent to Eq. (3) for the single-layer CPA. We obtained also the input beam parameters; the irradiance ratio and phase shift between the two input beams were determined from Eqs (9) and (10) to be 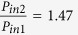 and *φ*_12_ = 0°, respectively.

If we apply Eq. (22) for this ENZ CPA at 1424 nm (TM wave), we obtain the subwavelength waveguide characteristics of ENZ CPA as follows; the vertical wavevector of ENZ CPA is *κ*_*f*_ = (0.55 − *i*6.15) × 10^6^ *m*^−1^, the order is *m* = 0, and the effective index of a propagating ENZ CPA wave is *N*_*eff*_ = 1.41. We find that the electric field decreases rapidly in the transverse direction, and the ultrathin subwavelength thickness obtained from the admittance matching of Eq. (16) is in good agreement with the zeroth order of Eq. (22), implying that ENZ CPA occurs due to critical coupling to a propagating wave in the longitudinal direction along the film.

**Figure 3:**
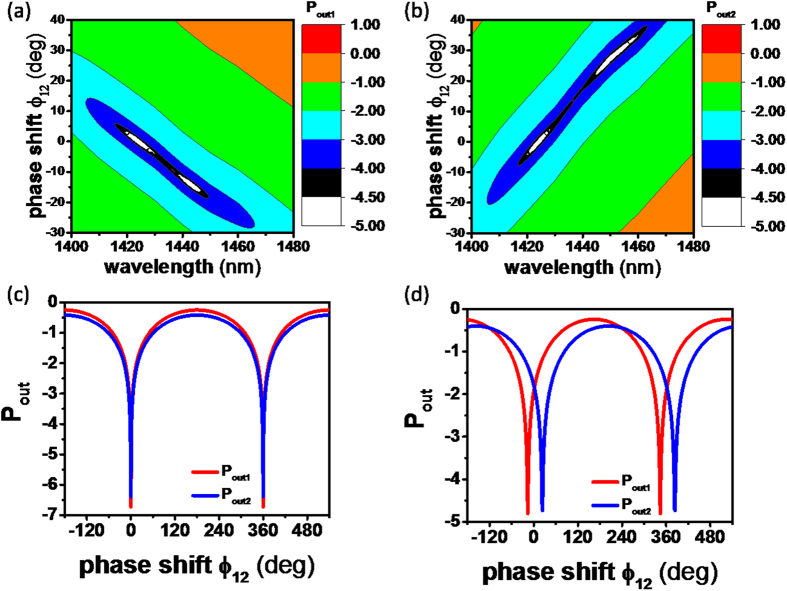
2D contour maps of (**a**) log(*P*_*out*1_) and (**b**) log(*P*_*out*2_) as functions of *φ*_12_ and wavelength. The color map is on the log scale. CPA occurs at 1424 nm, 0° and 1446 nm, −26° in *P*_*out*1_ and 1424 nm, 0° and 1446 nm, 24° in *P*_*out*2_. Calculated optical modulations of *P*_*out*1_ and *P*_*out*2_ on the log scale as a function of *φ*_12_ at wavelengths of (**c**) 1424 nm and (**d**) 1446 nm.

Now, we use this ENZ CPA for all-optical switching. The 2D contour maps of *P*_*out*1_ and *P*_*out*2_ are shown as functions of the relative phase shift *φ*_12_ and the incident wavelength in Fig. 3. Firstly, two-port CPA (i.e., *O*_1_ = *O*_2_ = 0) is observed at λ = 1424 nm, *φ*_12_ =0° in both Fig. 3(a,b). Secondly, one-port CPA with *O*_1_ = 0 occurs at λ = 1446 nm, *φ*_12_ = −16° in Fig. 3(a), whereas one-port CPA with *O*_2_ = 0 at λ = 1446 nm, *φ*_12_ = 24° in Fig. 3(b). The one-port CPA at 1446 nm can be explained as follows: Though the output irradiances are given by Eqs (11) and (12) due to 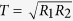 at 1446 nm as shown in Fig. 3(d), the phase change is either Δ_1_ = 0° or Δ_2_ = 0° depending on how the external phase shift *φ*_12_ is controlled.

The two-port CPA optical switch at 1424 nm is shown as a function of the phase shift *φ*_12_ in Fig. 3(c): the “ON” state (*O*_1_ = *O*_2_ = 0) is apparent at *φ*_12_ = 0°, and the optical switching effects are same in both ports. Optical switching behaviors at 1446 nm are also evident in Fig. 3(d). At *φ*_12_ = −16° the “ON” state (*O*_1_ = 0) appears in Port 1, whereas the “OFF” state (*O*_2_ ≠ 0) occurs in Port 2, while at *φ*_12_ = 24° the optical switch state is reversed. Equation (4) has two solutions at different wavelengths: one for two-port CPA and the other for one-port CPA. The optical switching phenomena in [Glass|ITO-1|ZnSe] CPA are summarized in Table 1.

### Broadband ENZ CPA in ITO Multilayers

**Figure 4:**
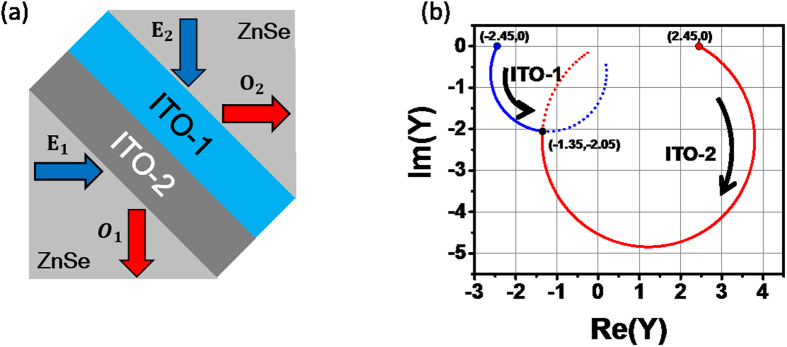
(**a**) Schematic diagram of broadband CPA device [ZnSe|ITO-1|ITO-2|ZnSe] and (**b**) modified admittance diagram of [ZnSe|ITO-1(14.22nm)|ITO-2(20.13 nm)|ZnSe] at 1550 nm in wavelength and with 45° incident angles, where *N*_*ITO*−1_ = 0.27 − *i*0.84, *N*_*ITO*−2_ = 0.59 − *i*0.30, and *n*_*ZnSe*_ = 2.45 were used.

Extending the previous ITO single-layer study, we now propose a broadband ENZ CPA system and apply it to multi-wavelength all-optical switching. As shown in Fig. 4(a), we consider an ITO double layer sandwiched between two ZnSe prisms (i.e. [ZnSe|ITO-1|ITO-2|ZnSe]), and fix the incidence angles as 45° in the both ZnSe prisms (*n*_0_ = *n*_*s*_ = 2.45). This configuration was chosen to realize near-IR broadband CPA while keeping the structure as simple as possible. We can broaden the bandwidth further by increasing the number of ITO layers. The ENZ wavelengths (i.e. *Re*(*ε*) = 0) of each ITO film are 1435 nm for ITO-1 and 1605 nm for ITO-2 (Fig. 1(b)). As we did in the previous section, we will first find CPA parameters graphically using the admittance matching diagram, and then compare them with the general CPA conditions derived from the scattering matrix method.

The admittance diagram shows that the forward modified admittance locus of ITO-1 (blue line) at a TM-wave wavelength of 1550 nm starts at (−2.45, 0) in Fig. 4(b), while the backward modified admittance (red line) begins at (+2.45, 0). The intersection of two loci of ITO-1 and ITO-2 at (−1.35, −2.05) indicates ITO-1 and ITO-2 thicknesses of 14.22 nm and 20.13 nm, respectively. Furthermore, the irradiance ratio and phase shift between the two input beams were determined from Eqs (9) and (10) as 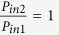 and *φ*_12_ = 1°, respectively. This method is a simple and intuitive means of determining the structural parameters in Eq. (15) for ENZ thin films exhibiting CPA, such as the thin-film materials, thicknesses, and the incident angles in media.

**Figure 5:**
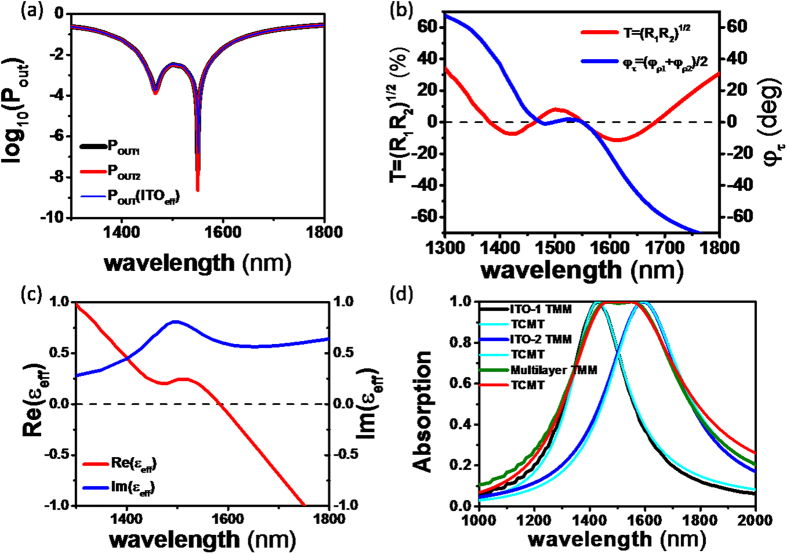
(**a**) Calculated output irradiances 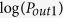 and log(*P*_*out*2_) of [ZnSe|ITO-1|ITO-2|ZnSe] at near IR wavelengths. 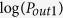 and log(*P*_*out*2_) of [ZnSe| Effective ITO |ZnSe] were also calculated. (**b**) Calculated (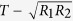) (red) and 
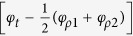
 (blue) of [ZnSe|ITO-1|ITO-2|ZnSe] as a function of wavelength; 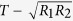 occurs at 1381 nm, 1463 nm, 1550 nm, and 1683 nm, whereas 
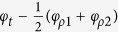
 occurs at 1474 nm, 1497 nm, and 1550 nm. (**c**) *ε*_*eff*_ of ITO-1 and ITO-2 layers calculated by using effective medium approximation. ENZ wavelength at Re(*ε*_*eff*_) = 0 is 1585 nm, and the broadband ENZ regime is evident at 1443–1576 nm. (**d**) Absorption spectra of [ZnSe|ITO-1(14.22nm)|ZnSe] and [ZnSe|ITO-2(20.13nm)|ZnSe] calculated by TMM and fitted by Lorentzian CPA spectrum of Eq. (S15 using TCMT in Supplementary Note B. Broad CPA spectrum of [ZnSe|ITO-1|ITO-2|ZnSe] calculated by TMM was also fitted by equivalent CPA spectrum using Eq. (S15) and two Lorentzian spectra.

The normalized output irradiances log(*P*_*out*1_) and log(*P*_*out*2_) were calculated and are shown in Fig. 5(a). The broadband range of CPA with >99% absorptance is apparent in 1443~1576 nm, and two CPA dips are evident at 1466 nm and 1550 nm in the CPA band. The CPA conditions were calculated using Eqs (4) and (5) and are plotted as a function of wavelength in Fig. 5(b). Four wavelengths with 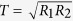 are evident: 1381 nm, 1463 nm, 1550 nm, and 1683 nm, and three wavelengths with 
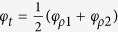
 are apparent: 1474 nm, 1497 nm, and 1550 nm. The band with 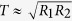 and 
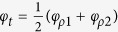
 is between 1443 and 1576 nm, which is consistent with the broad CPA band shown in Fig. 5(a). In addition, the CPA wavelength satisfying both CPA conditions simultaneously was found to be 1550 nm; perfect admittance matching occurs at this wavelength, as shown in Fig. 5(b).

Since the thicknesses of the ITO-1 and ITO-2 layers are much less than the wavelength 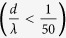, the effective dielectric constant (*ε*_*eff*_) of the ITO double layer can be calculated using the effective medium approximation^24^,^31^:


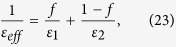


where *f* is the ratio of ITO-1 layer thickness to the total thickness. Figure 5(c) shows the real and imaginary parts of *ε*_*eff*_ for the two ENZ ITO layers. Its real part *Re*(*ε*_*eff*_) is close to zero over the broad spectral region from 1443–1576 nm, indicating that the broadband ENZ regime was obtained. Using *ε*_*eff*_, the CPA spectrum was calculated, and Fig. 5(d) shows that the broad ENZ wavelength region corresponds closely to the broad CPA regime. Again, broad CPA (i.e., radiative mode) occurs at broad *Re*(*ε*_*eff*_) slightly larger than zero and at shorter wavelength regime than the ENZ wavelength (1585 nm) of the effective layer at *Re*(*ε*_*eff*_) = 0.

Since ENZ absorption is caused by radiative resonant plasmons, Eq. (S23) of TCMT in Supplementary Note D can be applied to determining the damping constants of ENZ resonances at CPA. The absorption spectra of [ZnSe|ITO-1(14.22 nm)|ZnSe] and [ZnSe|ITO-2(20.13 nm)|ZnSe] calculated by TMM and fitted by TCMT are shown in Fig. 5(d).

It is evident that both methods yield the same CPA wavelengths at 1429 and 1594 nm. The broad CPA spectrum of [ZnSe|ITO-1|ITO-2|ZnSe] that was calculated by the TMM was also fitted by the sum of two Lorentzian spectra using Eq. (S15) in Supplementary Note B. We then obtained an equivalent CPA spectrum for [ZnSe|ITO-1|ITO-2|ZnSe], which is slightly narrower than the simple summation of the two spectra and which corresponds closely to that calculated by the TMM. Then, we determined the intrinsic and radiative damping constants of [ZnSe|ITO-1|ITO-2|ZnSe] structure to be Γ_*i*_ = Γ_*r*_ = 8.1 × 10^13^ rad/s in the broad CPA spectrum, indicating that the broadband ENZ CPA is the coupling of the two resonant CPAs in the ENZ ITO layers.

We also numerically simulated the same [ZnSe|ITO-1|ITO-2|ZnSe] structure, and verified that our analytic results agree well with the finite-difference time domain (FDTD) simulations. We show field profiles for our CPA structure too. For more details on numerical simulations, see Fig. S1 in Supplementary Information.

### Multi-wavelength All-optical Switching

**Figure 6:**
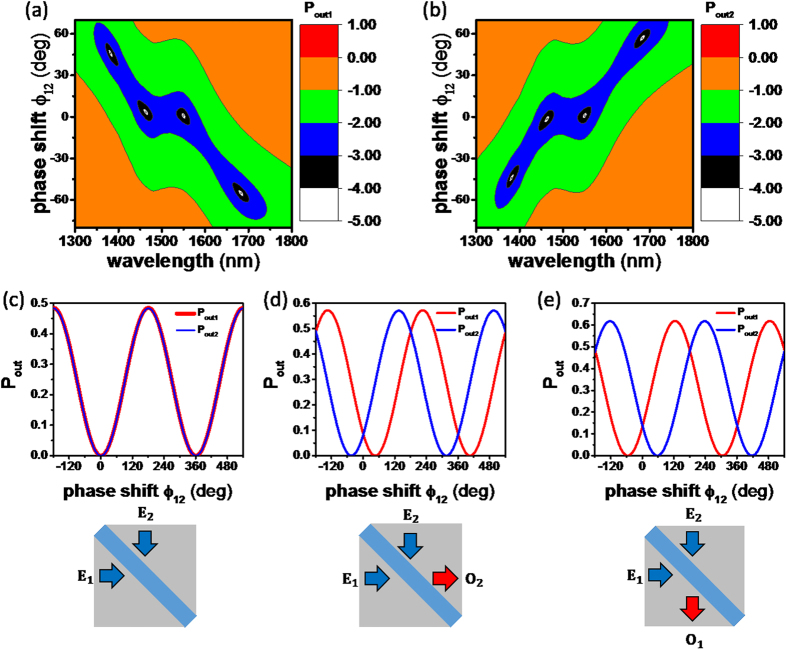
2D contour maps of (**a**) log(*P*_*out*1_) and (**b**) log(*P*_*out*2_) as a function of *φ*_12_. The color map is on the log scale. Optical switching of *P*_*out*1_ and *P*_*out*2_ at (**c**) 1550 nm, (**d**) 1381 nm, and (**e**) 1683 nm as a function of *φ*_12_.

Finally, we investigate the optical switching of the broadband CPA of [ZnSe|ITO-1|ITO-2|ZnSe]; *P*_*out*1_ and *P*_*out*2_ are shown on 2D contour maps as a function of the relative phase shift *φ*_12_ and incident wavelength in Fig. 6(a,b), respectively. Firstly, broadband CPA greater than 99% absorptance is observed in the 1443 ~ 1576 nm wavelength range at 1° phase shift (horizontal blue area, *O*_1_ = *O*_2_ = 0, i.e. two-port CPA) in Fig. 6(a,b); the bandwidth is ~133 nm in spectrum and the phase shift range is −5 ~ 7°. Secondly, the narrowband one-port CPAs are also evident: the one-port CPAs with *O*_1_ = 0 are found at λ = 1381 nm, *φ*_12_ = 46° and λ = 1683 nm, *φ*_12_ = −55° (tilted blue area) in Fig. 6(a), whereas the one-port CPAs with *O*_2_ = 0 are evident at λ = 1381 nm, *φ*_12_ = −44° and λ = 1683 nm, *φ*_12_ = 57° in Fig. 6(b).

The two-port broadband CPA optical switching at 1550 nm is shown as a function of *φ*_12_ in Fig. 6(c), and the “ON” state (*O*_1_ = *O*_2_ = 0) is apparent in both ports at *φ*_12_ = 1°. Similarly the broadband optical switching is evident at wavelengths of 1443~1576 nm at *φ*_12_ ≈ 1°. The optical switching of one-port CPA at 1381 nm and 1683 nm is shown in Fig. 6(d,e), respectively. At 1381 nm, the “ON” state with *O*_1_ = 0 appears at *φ*_12_ = 46°, whereas the “ON” state with *O*_2_ = 0 appears at *φ*_12_ = −44°. Similarly at 1683 nm, the “ON” state with *O*_1_ = 0 appears at *φ*_12_ = −55°, whereas the “ON” state with *O*_2_ = 0 appears at *φ*_12_ = 57°. Thus, the one-port optical switching of the narrowband CPA at 1381 nm and 1683 nm depends on the phase shift, and the two-port optical switching of the broadband CPA at 1443 ~ 1576 nm at *φ*_12_ = 1°. The multi-wavelength optical switching behaviors in narrowband and broadband [ZnSe|ITO-1|ITO-2|ZnSe] CPA device are summarized in Table 2.

We also investigated the incident angle dependence of the broadband CPA spectrum, which is shown in Fig. S2. As the incident angle increases from 44.5°–49°, the bandwidth between two CPA dips at 1466 nm and 1550 nm narrows, keeping the center wavelength middle in the band, until a single, merged broad resonance appears.

## Discussion

We want to clarify the difference between previous CPA proposals and our broadband CPA scheme. Previous CPA proposals were mostly based on nanostructured films. In those cases, elaborate multiple resonances in nanostructures may be employed to achieve broadband CPA. But, this can complicate the device structure and fabrication procedure. In contrast, we use simple, planar ENZ films, where optical properties can be easily controlled during the film growth (e.g. tuning film thicknesses and doping levels). Our approach simplifies the device fabrication and also facilitates the chip-scale integration with other optical components.

We notice that Ref. 8 also reported on CPA based on ultra-thin films. It studied two cases: Woltersdorff thickness and plasmon thickness. The former corresponds to a low-frequency region (DC to ~1 THz). The latter corresponds to the ENZ regime (i.e. *Re*(*ε*) = 0). Figure 6 in Ref. 8 shows strong absorption over a broad near-IR region in an ultra-thin tungsten film. But, as studied in Refs 23 and 32, normal-incidence perfect absorption in an ultra-thin film can happen only in a very lossy (large *Im*(*ε*) > 1) film. It is hard to achieve this in other low-loss (small *Im*(*ε*) < 1) materials. However, in our CPA scheme, perfect absorption can occur in a low-loss, ultra-thin ENZ film, thanks to critical coupling at the ENZ wavelength^23^,^24^. Moreover, this can be easily extended to broadband CPA using ENZ multilayer films. Therefore, ENZ materials provide a very general, flexible platform for high-frequency, broadband CPA. We believe that many CPA devices can benefit from our approach.

In summary, we proposed the broadband ENZ CPA system based on tunable ITO thin films, and investigated its application to multi-wavelength all-optical switching in the near-IR region. We derived general CPA conditions first, using the scattering matrix and admittance matching methods separately, and demonstrated their equivalence for thin-film CPA. Then, we designed a [ZnSe|ITO-1|ITO-2|ZnSe] structure exhibiting the broadband CPA at 45° incident angles in two ZnSe prisms. We found that the admittance matching method could extract not only the structural parameters, such as layer thicknesses and incident angles in media, but also input beam parameters, such as the power ratio and phase difference between two input beams. Finally, we analyzed multi-wavelength optical switching in our broadband ENZ CPA system. We can broaden the bandwidth further by increasing the number of ENZ layers. The same method can be also readily extended to other frequency ranges using different ENZ materials. Our proposal and theoretical analysis can provide design principles and guidelines for thin-film CPA devices, which can find various applications in optical switches, modulators, filters, sensors, and thermal emitters.

## Additional Information

**How to cite this article**: Kim, T. Y. *et al.* General Strategy for Broadband Coherent Perfect Absorption and Multi-wavelength All-optical Switching Based on Epsilon-Near-Zero Multilayer Films. *Sci. Rep.* **6**, 22941; doi: 10.1038/srep22941 (2016).

## Supplementary Material

Supplementary Information

## Figures and Tables

**Table 1:** Multi-wavelength optical switching in [Glass|ITO-1|ZnSe].

	Wavelength (nm)	Phase shift (*ϕ*_12_, degree)	*P*_*out*1_ (Port 1)	*P*_*out*2_ (Port 2)
Single- wavelength CPA	1424	0	**ON** (*P*_*out*1_ = 0, Δ_1_ = 0°)	**ON** (*P*_*out*2_ = 0, Δ_2_ = 0°)
	1446	−16	**ON** (*P*_*out*1_ = 0, Δ_1_ = 0°)	**OFF** (*P*_*out*2_ = 0.05, Δ_2_ = 40°)
		24	**OFF** (*P*_*out*1_ = 0.07, Δ_1_ = 40°)	**ON** (*P*_*out*2_ = 0, Δ_2_ = 0°)

If *P*_*out*_ = 0, the switch is defined as “ON.”

**Table 2:** Multi-wavelength optical switches in the broadband ENZ multilayer-ITO CPA device of [ZnSe|ITO-1|ITO-2|ZnSe] structure.

	Wavelength (nm)	Phase shift (*ϕ*_12_, degree)	*P*_*out*1_ (Port 1)	*P*_*out*2_ (Port 2)
Single- wavelength CPA	1381	−44°	**OFF** (*P*_*out*1_ = 0.29, Δ_1_ = −90°)	**ON** (*P*_*out*2_ = 0, Δ_2_ = 0°)
		46°	**ON** (*P*_*out*1_ = 0, Δ_1_ = 0°)	**OFF** (*P*_*out*2_ = 0.29, Δ_2_ = −90°)
	1683	−55°	**ON** (*P*_*out*1_ = 0, Δ_1_ = 0°)	**OFF** (*P*_*out*2_ = 0.43, Δ_2_ = 112°)
		57°	**OFF** (*P*_*out*1_ = 0.43, Δ_1_ = 112°)	**ON** (*P*_*out*2_ = 0, Δ_2_ = 0°)
Broadband CPA	1443–1576	1°	**ON** (*P*_*out*1_ = 0, Δ_1_ = 0°)	**ON** (*P*_*out*2_ = 0, Δ_2_ = 0°)
