# Supplementary material for: General Strategy for Broadband Coherent Perfect Absorption and Multi-wavelength All-optical Switching Based on Epsilon-Near-Zero Multilayer Films
Source: Sci Rep. 2016 Mar 11;6:22941. doi: 10.1038/srep22941 (PMC4786804; doi:10.1038/srep22941)
Supplement: Supplementary Information [file srep22941-s1.pdf]

Supplementary Information

General Strategy for Broadband Coherent Perfect Absorption and Multi-wavelength All-optical Switching Based on Epsilon-Near-Zero Multilayer Films

Tae Young Kim[1], Md. Alamgir Badsha[1], Junho Yoon[1], Seon Young Lee[1], Young Chul Jun[2], Chang Kwon Hwangbo[1*]

[1]Department of Physics, Inha University, Incheon 22212, Republic of Korea,

[2]School of Materials Science and Engineering, Ulsan National Institute of Science and Technology (UNIST), Ulsan 44919, Republic of Korea,

*hwangbo@inha.ac.kr

Supplementary Figure S1: FDTD simulations of ENZ CPA in [ZnSe|ITO-1|ITO-2|ZnSe].

Supplementary Figure S2: Angular dependence of broadband CPA in [ZnSe|ITO-1|ITO-2|ZnSe].

Supplementary Note A: Admittances and Scattering matrix elements.

Supplementary Note B: Critical coupling at the ENZ frequency

Supplementary Note C: Phase matching condition (Transverse resonance condition)

Supplementary Note D: Temporal coupled-mode theory (TCMT)

Reference:

Supplementary Figure S1

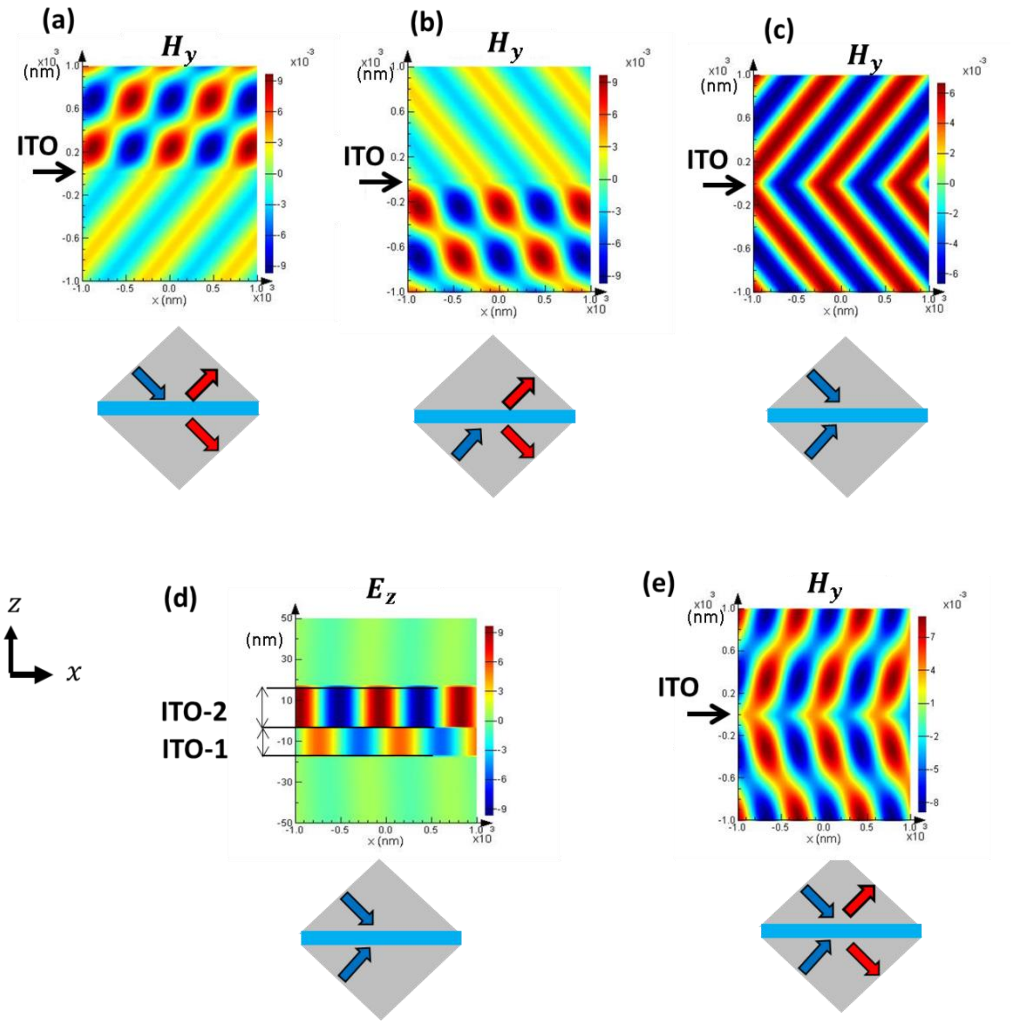

Fig. S1. The finite-difference time domain (FDTD) simulations of CPA in [ZnSe|ITO-1|ITO-2|ZnSe] [RS1]. The wavelength of incident light is fixed as 1550 nm. (a) A TM wave of E_1 is incident on [ZnSe|ITO-1|ITO-2|ZnSe] at an $45°$incident angle. The incident and reflected waves form an *interference pattern* in the incident medium (ZeSe), and the transmitted wave exits through the substrate (ZnSe). The magnetic field profile (H_y) is shown here. The reflectance is $R_1 = 25\%$, transmittance $T_1 = 25\%$, and absorptance $A = 50\%$. (b) It is the same as (a), but we only have E_2 at $45°$. Again, $R_2 = 25\%$, $T_2 = 25\%$, and $A = 50\%$. (c) If both E_1 and E_2 are present, CPA occurs and no transmitted light exist. Therefore, no interference patterns are observed, and only the incident plane waves are present in both incident and substrate media. It matches well with our analytic calculations in the main text. (d) The normal electric field profile (E_z) for the case (c). The field is strongly confined and enhanced in the ENZ multilayer. In both layers, the constructive interference patterns are observed, and it is in good agreement with Fig. 5(a) in the main text that shows CPA at 1550 nm. It also shows the transverse resonance condition in the ENZ multilayer is valid. (e) If the incident angles are $60°$, CPA does not occur and the interference patterns are present on both sides due to reflected and transmitted waves.

Supplementary Figure S2

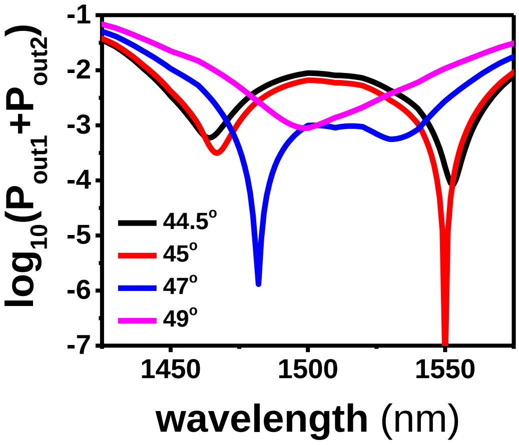

Fig. S2. Angular dependence of broadband CPA in [ZnSe|ITO-1|ITO-2|ZnSe]. As the incident angle increases from 44.5° to 49°, the bandwidth between two CPA dips at 1466 nm and 1550 nm narrows, keeping the center wavelength middle in the band, until a single, merged broad resonance appears.

Supplementary Note A: Admittances and Scattering matrix elements

At $E_1 = 1$ and $E_2 = 0$ in Fig. 1 of the main text, B and C at z_1 can be expressed, using the transfer matrix method, as

$$\begin{bmatrix} B \\ C \end{bmatrix} = \begin{bmatrix} \cos\delta & i\frac{\sin\delta}{\eta_f} \\ i\,\eta_f\,\sin\delta & \cos\delta \end{bmatrix} \begin{bmatrix} 1 \\ \eta_s \end{bmatrix}, \tag{S1}$$

where the admittances of TE and TM waves are given by

$$\eta_{0TE} = n_0 \cos\theta_0\,, \eta_{0TM} = \frac{n_0^2}{\eta_{0TE}}, \tag{S2}$$

$$\eta_{fTE} = (n - ik)\cos\theta\,, \eta_{fTM} = \frac{N_f^2}{\eta_{fTE}}, \tag{S3}$$

and

$$\eta_{sTE} = n_s \cos\theta_s\,, \eta_{sTM} = \frac{n_s^2}{\eta_{sTE}} \tag{S4}$$

in the incident medium (n_0), film ($N_f = n - ik$), and substrate (n_s), respectively (Ref. [26]). Since the reflection amplitude coefficient ρ_1 in Port 1 is given by

$$\rho_1 = \frac{\eta_0 - \frac{C}{B}}{\eta_0 + \frac{C}{B}}, \tag{S5}$$

we obtain

$$\rho_1 = \frac{\eta_s(\eta_0^2 - \eta_f^2)}{(\eta_0 + \eta_s)(\eta_0\eta_s + \eta_f^2)}. \tag{S6}$$

In deriving Eq. (S6), Eq. (16) was substituted into Eq. (S1), and B and C from Eq. (S1) were substituted into Eq. (S5). Additionally, the transmission amplitude coefficient τ_1 in Port 2 can be derived as

$$\tau_1 = \frac{2\eta_0}{\eta_0 B + C} = \pm\frac{\eta_0\sqrt{(\eta_0^2 - \eta_f^2)(\eta_s^2 - \eta_f^2)}}{(\eta_0 + \eta_s)(\eta_0\eta_s + \eta_f^2)}. \tag{S7}$$

Similarly, at $E_1 = 0$ and $E_2 = 1$, B and C at z_2 can be written as

$$\begin{bmatrix} B \\ C \end{bmatrix} = \begin{bmatrix} \cos\delta & i\frac{\sin\delta}{\eta_f} \\ i\,\eta_f\,\sin\delta & \cos\delta \end{bmatrix} \begin{bmatrix} 1 \\ \eta_0 \end{bmatrix}. \tag{S8}$$

Then, ρ_2 and τ_2 can be calculated respectively from Eqs. (16) and (S8) as

$$\rho_2 = \frac{\eta_s B - C}{\eta_s B + C} = \frac{\eta_0(\eta_s^2 - \eta_f^2)}{(\eta_0 + \eta_s)(\eta_0\eta_s + \eta_f^2)} = \frac{\eta_0(\eta_s^2 - \eta_f^2)}{\eta_s(\eta_0^2 - \eta_f^2)}\rho_1, \tag{S9}$$

and

$$\tau_2 = \frac{2\eta_s}{\eta_s B + C} = \pm\frac{\eta_s\sqrt{(\eta_0^2 - \eta_f^2)(\eta_s^2 - \eta_f^2)}}{(\eta_0 + \eta_s)(\eta_0\eta_s + \eta_f^2)} = \frac{\eta_s}{\eta_0}\tau_1. \tag{S10}$$

Then, it can be easily shown that Eqs. $(S6), (S7), (S9)$, and $(S10)$ satisfy Eq. (3), indicating that Eq. (16), which was derived from the admittance matching condition, is equivalent to the CPA condition obtained from the scattering matrix method. Furthermore, Eq. (3) can be rewritten as

$$\left(\rho_1 - \sqrt{\frac{\eta_0^2 - \eta_f^2}{\eta_s^2 - \eta_f^2}}\tau_2\right)\left(\rho_1 + \sqrt{\frac{\eta_0^2 - \eta_f^2}{\eta_s^2 - \eta_f^2}}\tau_2\right) = 0, \tag{S11}$$

indicating that two input beams should have even and odd phases.

Supplementary Note B: Critical coupling at the ENZ frequency

Since radiative resonant plasmon absorption occurs at the ENZ frequency, the elements of scattering matrix in Eq. (2) for an ENZ thin film in air (i.e., a film with a symmetric structure) can be obtained around the ENZ frequency using the Drude model. For an ultrathin ENZ film, we present the derivation of the scattering matrix elements of Eq. (2) by the TMM. In the case of p-polarized light, B and C at an oblique incidence θ_0 can be written as

$$\begin{bmatrix} B \\ C \end{bmatrix} = \begin{bmatrix} \cos\delta & i\frac{\sin\delta}{\eta_f} \\ i\eta_f\sin\delta & \cos\delta \end{bmatrix}\begin{bmatrix} 1 \\ \eta_s \end{bmatrix} \cong \begin{bmatrix} 1 & i\frac{\delta\cos\theta}{\sqrt{\varepsilon}} \\ i\frac{\sqrt{\varepsilon}}{\cos\theta}\delta & 1 \end{bmatrix}\begin{bmatrix} 1 \\ \frac{1}{\cos\theta_0} \end{bmatrix}, \tag{S12}$$

where $\eta_f = \frac{\sqrt{\varepsilon}}{\cos\theta}$ is the tilted admittance of the film, θ is the transmission angle in the film, and $\sqrt{\varepsilon} = N_f$ is the complex refractive index of the film. For an ultrathin ENZ film, the optical phase thickness can be approximated as $\delta = \frac{2\pi}{\lambda}\sqrt{\varepsilon}d\cos\theta \ll 1$. Then, the reflection and transmission coefficients can be derived as

$$\rho = -\frac{B - C\cos\theta_0}{B + C\cos\theta_0} \approx \frac{i\frac{\omega d}{c}\sin\theta_0\tan\theta_0}{2\varepsilon - i\frac{\omega d}{c}\sin\theta_0\tan\theta_0} \approx \frac{i\Gamma_r}{(\omega-\omega_e) - i(\Gamma_i+\Gamma_r)} \tag{S13}$$

and

$$\tau = \frac{2}{B + C\cos\theta_0} \approx \frac{2\varepsilon}{2\varepsilon - i\frac{\omega}{c}d\sin\theta_0\tan\theta_0} \approx \frac{(\omega-\omega_e) - i\Gamma_i}{(\omega-\omega_e) - i(\Gamma_i+\Gamma_r)}, \tag{S14}$$

where $\Gamma_i = \frac{1}{2}\omega_e Im(\varepsilon)$ is the intrinsic damping constant, $\Gamma_r = \frac{1}{4}\omega_e k_e d\sin\theta_0\tan\theta_0$ is the radiative damping constant, d is the thickness of the film and θ_0 is the incident angle of p-polarized light. In deriving Eqs. (S13) and (S14), the Drude model approximation was employed at the ENZ frequency $\omega \cong \omega_e$.

For bidirectional input beams, the CPA spectrum for an ENZ film is given by

$$A_{CPA}(\omega) = 1 - |\rho + \tau|^2 = \frac{4\Gamma_i\Gamma_r}{(\omega-\omega_e)^2 + (\Gamma_i+\Gamma_r)^2}, \tag{S15}$$

while for a unidirectional input beam, the absorption spectrum is given by

$$A(\omega) = 1 - |\rho|^2 - |\tau|^2 = \frac{2\Gamma_i\Gamma_r}{(\omega-\omega_e)^2 + (\Gamma_i+\Gamma_r)^2}. \tag{S16}$$

The maximum absorption for bidirectional input beams is $A_{CPA}(\omega_e) = 1$ when the critical coupling condition $\Gamma_i = \Gamma_r$ (i.e., $\frac{d}{\lambda} = \frac{Im(\varepsilon)}{\pi\sin\theta_0\tan\theta_0}$) is satisfied, while the maximum absorption for a unidirectional input beam is $A(\omega_e) = 0.5$ when $\Gamma_i = \Gamma_r$.

Equations (S13) and (S14) are the same as those obtained from the temporal coupled-mode theory (TCMT) for a resonant system at $r_d = 0$ and $t_d = 1$ in a symmetric system, as shown below in Supplementary Note **C**.

Supplementary Note C: Phase matching condition (Transverse resonance condition)

The admittance matching of Eq. (16) at CPA can be written as

$$\delta = \tan^{-1}\left(f_0\frac{(-i\eta_{0TE})}{\eta_{fTE}}\right) + \tan^{-1}\left(f_s\frac{(-i\eta_{sTE})}{\eta_{fTE}}\right) + m\pi, \tag{S17}$$

where

$$f_0 = \begin{cases} 1 \text{ for TE wave} \\ \frac{N_f^2}{n_0^2} \text{ for TM wave} \end{cases}, \tag{S18}$$

and

$$f_s = \begin{cases} 1 \text{ for TE wave} \\ \frac{N_f^2}{n_s^2} \text{ for TM wave} \end{cases}. \tag{S19}$$

If we define the normal wavevectors in film, incident medium, and substrate as $\kappa_f = \eta_{fTE}K_0 = N_f K_0 \cos\theta_f$, $\gamma_0 = -i\eta_{0TE}K_0$, and $\gamma_s = -i\eta_{sTE}K_0$, respectively, Eq. (S19) can be simplified as the phase matching condition for coherent perfect absorption:

$$2\kappa_f d = 2\varphi_0 + 2\varphi_s + 2m\pi, \tag{S20}$$

where $2\delta = 2\kappa_f d, 2\varphi_0 = 2\tan^{-1}\left(f_0 \frac{\gamma_0}{\kappa_f}\right), 2\varphi_s = 2\tan^{-1}\left(f_s \frac{\gamma_s}{\kappa_f}\right)$.

Supplementary Note D: Temporal coupled-mode theory (TCMT)

The resonance phenomena of nanostructures can be explained by TCMT [RS2-RS4]. Since the perfect absorption of an ITO thin film at the ENZ frequency is the resonant radiative plasmon absorption, it can be assumed that the direct matrix elements of an ENZ ITO thin film in TCMT are $r_d = 0$ and $t_d = 1$ for a transmission-type film [RS2, RS3]. Then, the scattering matrix can be written using TCMT as

$$[S] = \frac{-1}{i(\omega-\omega_e)+\Gamma_i+\Gamma_r}\begin{bmatrix} -\Gamma_r & i(\omega-\omega_e)+\Gamma_i \\ i(\omega-\omega_e)+\Gamma_i & -\Gamma_r \end{bmatrix}. \tag{S21}$$

The reflection and transmission coefficients in Eq. (S21) are the same as Eqs. (S13) and (S14), which were derived using the TMM.

Reference:

RS1. Lumerical FDTD Solutions software (2015). Lumerical Inc, Vancouver, Canada. URL https://www.lumerical.com/.

RS2. Fan, S., Suh, W. & Joannopoulos, J. D. Temporal coupled-mode theory for the Fano resonance in optical resonators. *J. Opt. Soc. Am. A* **20**, 569-572(2003).

RS3. Suh, W. & Fan, S. Temporal coupled-mode theory and the presence of non-orthogonal modes in lossless multimode cavities. *IEEE J. Quant. Electron.* **40**, 1511-1518 (2004).

RS4. Haus, H. A. in Waves and Fields in Optoelectronics, Ch 7, 200-216 (Prentice-Hall, 1984).
